# DNA ploidy and PTEN as biomarkers for predicting aggressive disease in prostate cancer patients under active surveillance

**DOI:** 10.1038/s41416-024-02780-x

**Published:** 2024-07-03

**Authors:** Karolina Cyll, Erik Skaaheim Haug, Manohar Pradhan, Ljiljana Vlatkovic, Birgitte Carlsen, Sven Löffeler, Wanja Kildal, Karin Skogstad, Frida Hauge Torkelsen, Rolf Anders Syvertsen, Hanne A. Askautrud, Knut Liestøl, Andreas Kleppe, Håvard E. Danielsen

**Affiliations:** 1 Institute for Cancer Genetics and Informatics, Oslo University Hospital, 0424 Oslo, Norway; 2 Department of Urology, Vestfold Hospital Trust, 3103 Tønsberg, Norway; 3 Department of Pathology, Vestfold Hospital Trust, 3103 Tønsberg, Norway; 4 Department of Informatics, University of Oslo, 0316 Oslo, Norway; 5 Centre for Research-based Innovation Visual Intelligence, UiT The Arctic University of Norway, Tromsø, Norway; 6 Nuffield Division of Clinical Laboratory Sciences, University of Oxford, Oxford, OX3 9DU UK

**Keywords:** Tumour biomarkers, Tumour heterogeneity, Genomic instability, Prostate cancer, Tumour-suppressor proteins

## Abstract

**Background:**

Current risk stratification tools for prostate cancer patients under active surveillance (AS) may inadequately identify those needing treatment. We investigated DNA ploidy and PTEN as potential biomarkers to predict aggressive disease in AS patients.

**Methods:**

We assessed DNA ploidy by image cytometry and PTEN protein expression by immunohistochemistry in 3197 tumour-containing tissue blocks from 558 patients followed in AS at a Norwegian local hospital. The primary endpoint was treatment, with treatment failure (biochemical recurrence or initiation of salvage therapy) as the secondary endpoint.

**Results:**

The combined DNA ploidy and PTEN (DPP) status at diagnosis was associated with treatment-free survival in univariable- and multivariable analysis, with a HR for DPP-aberrant vs. DPP-normal tumours of 2.12 (*p* < 0.0001) and 1.94 (*p* < 0.0001), respectively. Integration of DNA ploidy and PTEN status with the Cancer of the Prostate Risk Assessment (CAPRA) score improved risk stratification (c-index difference = 0.025; *p* = 0.0033). Among the treated patients, those with DPP-aberrant tumours exhibited a significantly higher likelihood of treatment failure (HR 2.01; *p* = 0.027).

**Conclusions:**

DNA ploidy and PTEN could serve as additional biomarkers to identify AS patients at increased risk of developing aggressive disease, enabling earlier intervention for nearly 50% of the patients that will eventually receive treatment with current protocol.

## Introduction

Prostate cancer (PCa) is the second most common cancer in men worldwide, with over 1.4 million new cases in 2020 [1]. About 80% of cases are localized, and categorized into low-, intermediate-, and high-risk groups based on prostate-specific antigen (PSA) levels, tumour stage, and Gleason grade group (GGG) [2]. Presently, 40–74% of low-risk and 5–17% of intermediate-risk patients opt for active surveillance (AS) over immediate treatment with radical prostatectomy (RP) or radiotherapy [3–5]. AS aims to minimize overtreatment and treatment-associated side effects among patients with less aggressive tumours. Patients enroled in AS are regularly monitored with prostate biopsies, PSA measurements, rectal exams and, if available, magnetic resonance imaging [6]. Curative treatment is only recommended for those who exhibit signs of disease progression. A recent update from the ProtecT trial concluded that AS was safe for most low-risk and selected intermediate-risk patients. However, 9.4% of patients developed metastases during the 15-year follow-up [7].

The current criteria for inclusion and transition to treatment in AS protocols solely rely on standard clinicopathologic characteristics [6], which inadequately identify tumour aggressiveness in some patients [7]. Incorporating biomarkers that complement these characteristics to predict aggressive disease could improve AS utilization and better inform treatment decisions.

Several studies have investigated the prognostic significance of tissue-based molecular biomarkers in AS settings, mainly involving commercial gene expression classifiers [8–11]. However, these studies do not take into account tumour heterogeneity by relying on a single biopsy core obtained at diagnosis, and do not determine if adding these biomarkers to standard risk assessment tools improves risk evaluation. Furthermore, several of these studies were susceptible to bias, as treatment decisions may have been influenced by the biomarker’s assessment [10].

The progression of PCa is, at least partly, driven by genomic instability—a state in which cells accumulate genetic alterations rapidly [2]. Genomic instability can be induced by various mechanisms, resulting in abnormal DNA content and tumour heterogeneity, both linked to a more aggressive cancer phenotype [12, 13]. DNA ploidy is a marker of large-scale genomic instability and has repeatedly demonstrated prognostic value in PCa (reviewed in [13, 14]). The PTEN tumour suppressor is another relevant prognostic biomarker for PCa, particularly for low- and intermediate-risk patients (reviewed in [15]). PTEN loss is a common genomic alteration in PCa, disrupting multiple pathways and contributing to genomic instability [15]. In a previous study [16], we demonstrated that combining these biomarkers could improve risk stratification following RP and hypothesized that a similar approach could also improve decision-making in a preoperative setting.

In this study, we retrospectively analysed DNA ploidy and PTEN expression in a community-based AS cohort of PCa patients at low or intermediate risk according to standard clinicopathological parameters at diagnosis. To address tumour heterogeneity and potential sampling errors, we assessed both biomarkers in all available tumour specimens at diagnosis and during AS.

## Methods

### Study cohort and sample processing

Between August 2003 and December 2020, 712 patients diagnosed with PCa were prospectively enroled in the AS programme at Vestfold Hospital Trust, Norway. Criteria for AS inclusion and discontinuation, as well as monitoring with PSA tests, rectal examinations, biopsies and multiparametric magnetic resonance imaging (MRI) are summarized in Table 1. The decision to include or discontinue AS for all patients was based on recommendations from a multidisciplinary team and shared decision-making between patients and their clinicians. At diagnosis, patients were classified as low-risk (CAPRA score 0–2 and PSA < 10 ng/ml and GGG 1) or intermediate-risk (CAPRA score 3–5 and/or PSA 10–20 ng/ml and/or GGG 2). Tissue material was collected until May 2022, and follow-up data until May 2023.

**Table 1:** Active surveillance protocol at Vestfold Hospital Trust.

AS inclusion criteria	Follow-up scheme	AS discontinuation criteria
• Age <75 years^a^ • GGG < 3 • PSA ≤ 20 ng/ml • cT <3 • Life expectancy >5 years^b^ • Patient preference **Low risk** CAPRA^c^ 0–2 and PSA < 10 ng/ml and GGG 1 **Intermediate risk** CAPRA^c^ 3–5 and/or PSA 10–20 ng/ml and/or GGG 2	**Low risk** • PSA every 3 months during the first 2 years and every 6 months thereafter • Repeat biopsy 12 months after AS enrolment and every 60 months thereafter (or at increase of PSA level or tumour size) • MRI† after 12, 48 and 60 months **Intermediate risk** • PSA every 3 months during the first 2 years and every 6 months thereafter • Repeat biopsy 12 and 24 months after AS enrolment, and every 60 months thereafter (or at increase of PSA level or tumour size) • MRI after 12, 24 and 48 months, and every 24 months after that	**Triggers for treatment** • Histological reclassification (GGG ≥ 3 or increase in number of positive biopsy cores)^d^ • Biochemical reclassification (PSA > 20 ng/ml or PSA doubling time <1 year)^e^ • Clinical reclassification (cT ≥3) • Radiological reclassification (any indication of “progressing appearances” such as increased overall PI-RADS score, new MRI-visible areas, increasing lesion size, EPE or SVI)^d^ • Patient preference^f^ **Transferal to watchful waiting** • Age ≥75^a^ • Life expectancy ≤5 years^b^

*AS* active surveillance, *CAPRA* Cancer of the Prostate Risk Assessment, *EPE* extraprostatic extension, *GGG* Gleason grade group, *MRI* magnetic resonance imaging, *PI-RADS* Prostate Imaging Reporting and Data System, *PSA* prostate-specific antigen, *SVI* seminal vesicle invasion.

^a^Increased to 80 years in 2014.

^b^Based on Charlson comorbidity index.

^c^If available.

^d^As determined by the multidisciplinary team.

^e^On at least two consecutive measurements. PSA reclassification was not considered as a sole reason for treatment after 2018.

^f^Patients treated by personal preference were excluded from the study.

**Fig. 1:**
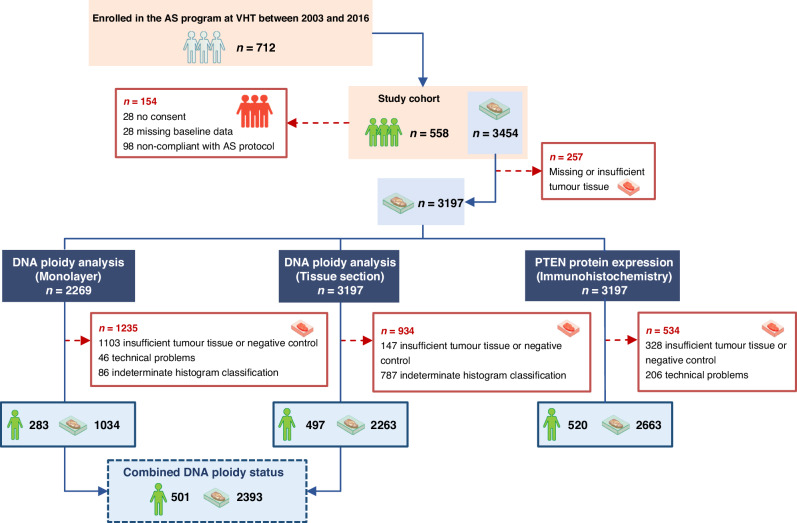
Overview of analyzed patient material and methods. Out of the 712 patients, 154 were excluded for the following reasons: lack of consent (*n* = 28), missing baseline data (*n* = 28), or non-compliance with AS protocol inclusion criteria (*n* = 44) or AS termination criteria (no treatment despite protocol-based disease reclassification (*n* = 43), or treatment due to personal choice without protocol-based disease reclassification (*n* = 11)). Among the 558 patients included, a total of 1102 biopsies and 109 TURP procedures were performed, yielding at least 3454 tumour-containing tissue blocks (for 26 procedures, the exact number of tumour-containing tissue blocks was unknown and was assumed to be “1”). Out of these, at least 257 tissue blocks were either from procedures performed after material collection had terminated (May 2022), but before follow-up data was collected (May 2023), were missing from the archive or had no remaining tumour tissue. A total of 3197 tumour-containing tissue blocks from 1012 biopsies and 103 TURP procedures were scheduled for DNA ploidy and PTEN immunohistochemistry analyses. DNA ploidy analysis using monolayers was attempted in 2269 cases, while DNA ploidy analysis using tissue sections was performed on all 3197 tissue blocks. A combined DNA ploidy status was determined using both monolayers and tissue sections, with preference given to monolayer DNA ploidy if available; otherwise, tissue section DNA ploidy was used. AS active surveillance, TURP transurethral resection of the prostate, VHT Vestfold Hospital Trust.

Of the 712 patients, 154 (22%) were excluded due to either lack of consent (*n* = 28), missing baseline data (*n* = 28), not meeting AS inclusion criteria (*n* = 44) or not being treated following disease reclassification (*n* = 54) (Fig. 1). The 558 included patients underwent 1371 (median 3 per patient; interquartile range (IQR) 2–4) needle biopsy procedures. Among these, 729 were systematic, 339 were targeted, 292 were a combination of both methods, and 11 procedures were of an unknown type. Out of the 558 patients, 105 had 109 transurethral resection of the prostate (TURP) procedures, with 98 performed at diagnosis. Tumour was diagnosed in 1211 (median 2 per patient; IQR 2–3) procedures, comprising 3454 (median 4 per procedure; IQR 2–5) formalin-fixed paraffin-embedded tissue blocks (each biopsy core was placed in one block).

Routine Gleason scoring was performed using the 2005 International Society of Urological Pathology (ISUP) or the 2014 ISUP guidelines [17, 18]. Available diagnostic histology slides were retrospectively reviewed by an experienced uropathologist (LV) using the 2014 ISUP guidelines [18].

Retrospective evaluation of DNA ploidy by image cytometry and PTEN protein expression by immunohistochemistry was performed in all tumour-containing blocks, except for 257 (7%) blocks that could not be collected or had no remaining tumour tissue (Fig. 1). DNA ploidy was examined in nuclear monolayers and tissue sections. For monolayer preparation, one to three 50 µm sections were obtained from 2269 blocks deemed to have an adequate tissue amount. For DNA ploidy assessment in tissue sections, one to three 5 µm sections were cut from all 3197 blocks. A single 3 µm section was cut from each of the 3197 blocks for PTEN immunohistochemistry.

### DNA image cytometry

Nuclear monolayers were prepared according to a modified Hedley’s method [19]. Identification of representative epithelial and stromal (reference) nuclei and DNA ploidy histogram classification was performed automatically using the PWS Classifier software (Room4 Ltd, Sussex, UK), as previously described [16]. However, the amount of tumour in 2031 blocks was insufficient for monolayer preparation, so a method for image cytometry on tissue sections was developed (see the Supplementary information for details) and applied to all tumour specimens. The agreement between DNA ploidy status measured using both methods was good (κ = 0.685; *p* < 0.001; 838 of 904 blocks (93%) had equal ploidy classification). A combined DNA ploidy status was determined using both monolayers and tissue sections, with priority given to monolayer DNA ploidy when available. DNA ploidy was analysed as a dichotomous biomarker with categories diploid and non-diploid (i.e. tetraploid or aneuploid). For the determination of DNA ploidy status at the procedural level, procedures containing both diploid and non-diploid blocks were classified as non-diploid.

### PTEN immunohistochemistry and scoring

Immunohistochemistry was performed after heat-induced epitope retrieval with a validated antibody against PTEN (rabbit monoclonal; clone 138G6, Cat# 9559, RRID:AB_390810, 1:400, Cell Signalling Technology, Danvers, MA) [20, 21], as previously described [22]. PTEN expression was scored independently in 10%-intervals by two observers, blinded to clinical, pathological and outcome data. Cancer cells were considered PTEN-negative if cytoplasmic and nuclear staining was absent or decreased compared with internal positive controls (benign glands and/or stroma). PTEN status was considered “lost” if ≤90% of the cells were PTEN-positive [23, 24]. The inter-observer agreement was high (κ = 0.831; *p* < 0.001; 2913 of 3066 blocks (95%) had equal PTEN status). A consensus score was used in further analyses. For procedures where PTEN was successfully measured in multiple blocks, we calculated the average PTEN score across all the blocks. This average score was then thresholded to determine the PTEN status at the procedural level.

### Endpoints

The primary and secondary endpoints for analysis were predetermined. The primary endpoint was treatment due to disease reclassification (Table 1), and this was determined for all included patients. Treatment-free survival (TFS) was defined as the time from the date of diagnosis to the date of AS discontinuation, or until end of follow-up (30th April 2023). The secondary endpoint was treatment failure, and this was determined for patients treated with RP or radiotherapy. Treatment failure-free survival (TFFS) was defined as the time from the completion of treatment to the first occurrence of biochemical recurrence or initiation of salvage radiotherapy, or until follow-up end. Biochemical recurrence was defined as PSA level of ≥0.2 ng/ml at least 6 weeks post-RP or a ≥2 ng/mL rise above nadir PSA post-radiotherapy [25].

### Statistical analyses

DNA ploidy and PTEN status were analysed both as individual biomarkers and as a dichotomous biomarker with categories: DNA ploidy and PTEN “normal” (DPP-normal) including only diploid and PTEN present tumours, and DNA ploidy or PTEN “aberrant” (DPP-aberrant) including tumours with either non-diploidy, PTEN loss or both aberrations. Biomarkers were evaluated using both time-invariant cause-specific analyses of TFS and TFFS, and time-dependent cause-specific analyses of TFS. Time-invariant analyses included biomarker status assessed at a single time point, and survival curves were visualized with the Kaplan-Meier method. Time-dependent analyses included biomarker status across all procedures except those indicating histological reclassification according to the AS protocol and those performed after any reclassification event. The aim was to assess biomarker performance before disease reclassification, while also accounting for measurements taken after the diagnosis. These analyses addressed variables changing over time, at each time point set to indicate the most aggressive disease status up to that point. Survival curves were depicted using the Simon-Makuch method, a Kaplan-Meier adaptation for situations where a time-dependent covariate defines patient groups [26]. To compare survival distributions, log-rank test was used for univariable analyses of categorical variables, while Wald’s chi-squared test was used for univariable analyses of continuous variables and multivariable analyses. Two multivariable analyses were performed using variables assessed at diagnosis and found to be significant in univariable analysis of TFS. The proportional-hazards assumption was evaluated using Schoenfeld residuals and found acceptable. DNA ploidy and PTEN status were integrated with the CAPRA score by adding 1 point if non-diploid and 1 if PTEN loss. A two-sided p-value was calculated to test the difference in the c-index between the standard and updated CAPRA score, based on 1 minus the confidence level of the largest bias-corrected and accelerated confidence interval (CI) that did not encompass 0. DNA ploidy and PTEN status performance for one versus all blocks was evaluated by randomly selecting one block per procedure 10,000 times and comparing marker performance for these 10,000 samples to the marker performance with all blocks using the one-sample *t* test. The effect of centralized review-based GGG on the performance of the combined DNA ploidy and PTEN biomarker was tested with a multivariable model including both markers and their interaction term by applying Wald’s chi-squared test to the interaction term. Associations were evaluated using Fisher’s exact test and Mann-Whitney *U* test. Concordance between dichotomized PTEN scores from two observers and DNA ploidy status in monolayers and tissue sections was measured using Cohen’s kappa. Two-sided *p* values < 0.05 were considered statistically significant. Calculations were performed using Stata/SE 17.0 (StataCorp, College Station, TX). Results are reported according to the Reporting Recommendations for Tumour Marker Prognostic Studies (REMARK, Supplementary Table S1) [27].

## Results

### Study cohort

Table 2 presents a summary of the clinicopathological characteristics of the included patients. Among the 558 patients, the median age was 65 (IQR 61-70), with 283 (51%) classified as low-risk and 275 (49%) as intermediate-risk at diagnosis.

**Table 2:** Patient characteristics at prostate cancer diagnosis.

Characteristic	All patients	Low-risk	Intermediate-risk
Patients	558	283	275
Age (yr), median (IQR)	65 (61–70)	64 (59–68)	67 (63–70)
PSA (ng/ml), n (%)			
≤6	237 (42)	173 (61)	64 (23)
>6 and ≤10	228 (41)	110 (39)	118 (43)
>10 and ≤20	93 (17)	0	93 (34)
Prostate volume (ml), median (IQR)	41 (30–56)	41 (30–53)	42 (31–58)
Missing	52 (9)	34 (12)	18 (7)
PSA density (ng/ml/cm^3^), median (IQR)	0.16 (0.11–0.23)	0.13 (0.09–0.19)	0.19 (0.13–0.27)
Missing	52 (9)	34 (12)	18 (7)
Gleason grade group^a^, n (%)			
1	382 (68)	283 (100)	99 (36)
2	176 (32)	0	176 (64)
Gleason grade group^b^, n (%)			
1	230 (41)	152 (54)	78 (28)
2	257 (46)	102 (36)	155 (56)
3	25 (4)	6 (2)	19 (7)
4	13 (2)	4 (1)	9 (3)
5	3 (1)	0	3 (1)
Missing	30 (5)	19 (7)	11 (4)
MRI outcome			
No lesion	57 (10)	32 (11)	25 (9)
3	97 (17)	53 (19)	44 (16)
4	235 (42)	101 (36)	134 (49)
5	79 (14)	32 (11)	47 (17)
Missing	90 (16)	65 (23)	25 (9)
Clinical T stage, n (%)			
cT0/pT1	406 (73)	215 (76)	191 (69)
cT2	152 (27)	68 (24)	84 (31)
CAPRA score^c^, n (%)			
0–2	325 (58)	278 (98)	47 (17)
3–5	226 (41)	0	226 (82)
Missing	7 (1)	5 (2)	2 (1)
Diagnostic procedure type, n (%)			
Biopsy	460 (82)	225 (80)	235 (85)
TURP	98 (18)	58 (20)	40 (15)
Positive biopsy cores, n (%)	2.0 (1.0–4.0)	2.0 (1.0–2.0)	3.0 (1.0–4.0)
Missing	5 (1)	3 (1)	2 (1)
Percentage of positive biopsy cores (%), median (IQR)	20 (10–40)	17 (10–25)	30 (17–50)
Missing	7 (1)	5 (2)	2 (1)
Maximum tumour extent in biopsy cores (mm), median (IQR)	4.0 (2.0–7.0)	3.0 (1.3–5.0)	5.0 (2.8–8.0)
Missing	14 (3)	12 (4)	2 (1)

Due to rounding the numbers may not sum to 100%.

*CAPRA* Cancer of the Prostate Risk Assessment, *IQR* interquartile range, *MRI* magnetic resonance imaging, *PIRADS* Prostate Imaging Reporting and Data System, *PSA* prostate-specific antigen, *TURP* transurethral resection of the prostate.

^a^Routine Gleason scoring performed according to the 2005 or 2014 International Society of Urological Pathology guidelines.

^b^Centrally reviewed Gleason scoring performed according to the 2014 International Society of Urological Pathology guidelines.

^c^When computing CAPRA score for patients diagnosed with TURP, the percentage of positive biopsies was replaced by the tumour percentage in the TURP specimen; specifically, 0 points were assigned for <5% tumour and 1 point was assigned for ≥5% tumour. Computation of CAPRA score was performed using routinely assessed Gleason scores.

Out of the 558 patients, 339 (61%) discontinued AS (Supplementary Fig. S1); 230 received curative treatment, while 109 discontinued AS due to other reasons, mostly (75%) transferal to watchful waiting. The median follow-up was 3.2 years (IQR 2.9–3.5) for those who discontinued AS and 6.0 years (IQR 5.4–6.5) for those who remained on AS. The risk grouping at diagnosis was not significantly different for patients who were treated and other patients (*p* = 0.18).

Treatment was initiated after histological reclassification in 159 (69%) patients and after clinical, radiological and/or biochemical reclassification in the remaining 71 (31%) of the 230 patients. The median time to treatment was 3.1 years (IQR 2.6–3.4). Out of the 230 treated patients, 136 (59%) were intermediate-risk, and they had significantly shorter TFS compared to low-risk patients, with median TFS of 2.2 vs. 4.4 years (hazard ratio (HR) 2.31; 95% CI 1.77–3.02; *p* < 0.001).

Post-treatment follow-up data were available for 219 patients. Among these, 47 out of 179 patients (26%) experienced treatment failure following RP, while 3 out of 40 patients (8%) experienced it after radiotherapy. The median post-treatment follow-up was 1.1 years (IQR 0.6–1.9) for those with treatment failure and 5.6 years (IQR 5.1–6.8) for those without.

### DNA ploidy and PTEN status

Non-diploid tumours were detected in 167 (33%) of the 501 patients (23% of procedures), while PTEN loss was observed in 118 (23%) of the 520 patients (16% of procedures). Both biomarkers were assessed in 493 patients (943 procedures). Non-diploid and PTEN loss tumours were observed in 47 (10%) patients (5% of procedures) (Supplementary Table S2).

Overall, DPP-aberrant tumours were observed in 221 (45%) of the 495 patients with a valid measurement, and their proportion was higher in intermediate-risk compared to low-risk patients (131 (51%) of 255 vs. 90 (39%) of 240; *p* = 0.0021). At diagnosis, the combined DNA ploidy and PTEN status was associated with GGG (*p* < 0.0001), cT stage (*p* = 0.036), CAPRA score (*p* = 0.0006), percentage of positive biopsy cores (*p* < 0.0001) and maximum cancer extent within a biopsy core (*p* < 0.0001), (Supplementary Table S3).

**Fig. 2:**
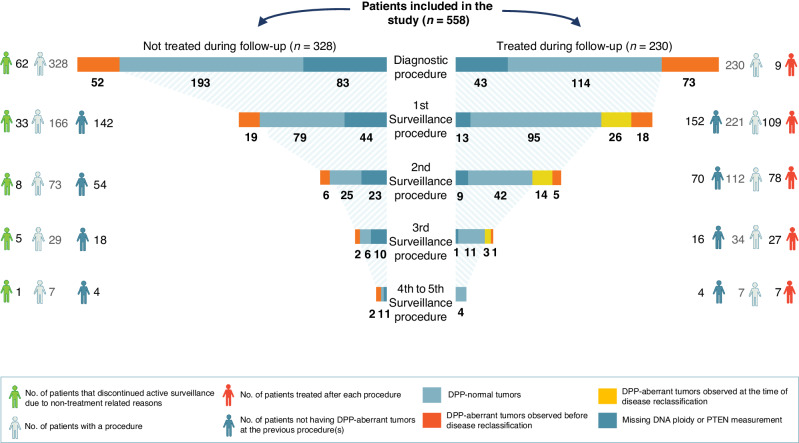
DNA ploidy and PTEN measurement results at each procedure, presented separately for treated and not treated patients. Among the 558 patients included in the study, 230 underwent treatment during the follow-up period, while 328 received no treatment. At the diagnostic procedure, 73 treated patients and 52 untreated patients were measured to have DNA ploidy and/or PTEN “aberrant” (DPP-aberrant) tumours (depicted by red bars). At the first surveillance procedure, DPP-aberrant tumours were observed in 44 treated patients and 19 untreated patients that did not demonstrate this characteristic at the previous procedure. Of the 44 treated patients with DPP-aberrant tumours in the first surveillance procedure, 26 also experienced disease reclassification based on standard clinicopathological parameters at that time (depicted by yellow bars). This is illustrated for all procedures. In total, DPP-aberrant tumours were observed in 140 (73 + 18 + 26 + 5 + 14 + 1 + 3) of the 226 treated patients (62%), and in 81 (52 + 19 + 6 + 2 + 2) of the 269 untreated patients (30%) with valid measurements. Among the treated patients, DPP-aberrant tumours were detected before disease reclassification in 97 (73 + 18 + 5 + 1) patients and at the time of disease reclassification in 43 (26 + 14 + 3) patients. DPP-normal DNA ploidy and PTEN “normal”, DPP-aberrant DNA ploidy and/or PTEN “aberrant”.

Figure 2 depicts DNA ploidy and PTEN measurement results at each procedure, separately for treated and untreated patients. DPP-aberrant tumours were more frequently observed in treated patients before disease reclassification than in untreated patients (97 (48%) of 202 vs. 81 (30%) of 269; *p* = 0.0001). This association was significant in both low-risk (31 (39%) of 79 vs. 37 (25%) of 148; *p* = 0.033) and intermediate-risk patients (66 (54%) of 123 vs. 44 (36%) of 121; *p* = 0.0071).

### Tumour heterogeneity

Tumour heterogeneity refers to the presence of variability or diversity within a tumour concerning various biomarkers. In our study, we assessed tumour heterogeneity in GGG, DNA ploidy, and PTEN status both individually and in combination, among different samples taken at each biopsy or TURP procedure from a given patient. Specifically, we found that 51% of patients exhibited tumour heterogeneity in GGG, 22% in DNA ploidy, 14% in PTEN status, and 31% in the combined biomarker at the diagnostic procedure. These percentages increased to 70%, 38%, 25% and 45% at the third surveillance procedures, respectively (Supplementary Table S4). Tumour heterogeneity was associated with treatment when evaluating GGG, PTEN and the combined biomarker at the diagnostic procedure (*p* = 0.048, *p* = 0.041 and *p* = 0.033, respectively), and when considering either biomarker at the last procedure before any treatment (*p* < 0.0001 for GGG, *p* = 0.0017, for DNA ploidy, *p* = 0.0057 for PTEN and *p* = 0.0009 for the combined biomarker), (Supplementary Table S5).

### DNA ploidy and PTEN status in analyses of treatment-free survival

DNA ploidy status was associated with TFS in univariable time-invariant (HR 1.90; *p* < 0.0001; Supplementary Fig. S2A) and time-dependent analysis (HR 1.91; *p* < 0.0001; Supplementary Fig. S2B). In the corresponding analyses of PTEN status, the HR was 1.98 (*p* < 0.0003; Supplementary Fig. S2C) and 2.05 (*p* < 0.0001; Supplementary Fig. S2D), respectively.

**Fig. 3:**
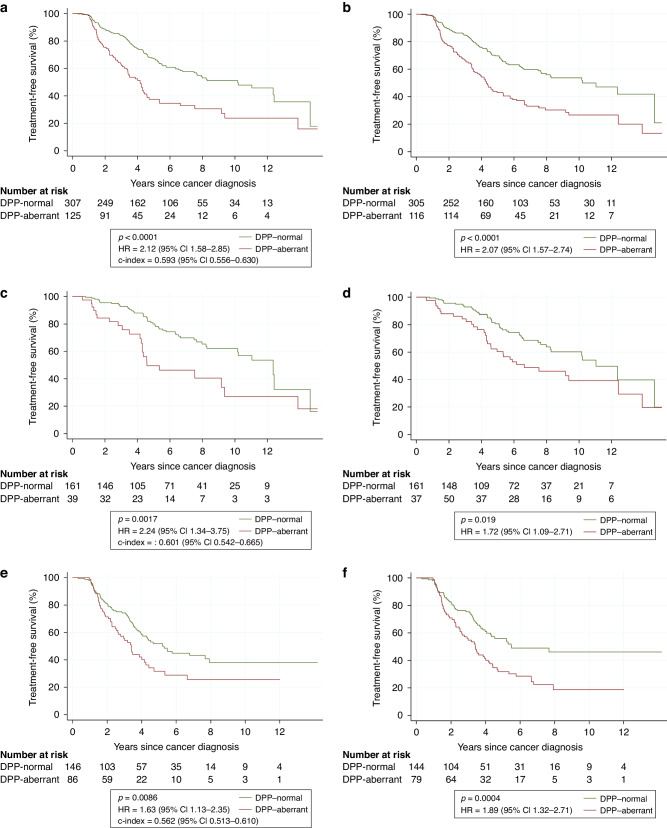
Analyses of treatment-free survival grouped by combined DNA ploidy and PTEN status. ** a**, **c**, **e** Time-invariant analyses. **b**, **d**, **f** Time-dependent analyses. **a**, **b** All patients. **c**, **d** Low-risk patients. **e**, **f** Intermediate-risk patients. CI confidence interval, DPP-normal DNA ploidy and PTEN “normal”, DPP-aberrant DNA ploidy and/or PTEN “aberrant”, HR hazard ratio.

The combined biomarker was associated with TFS in both univariable and multivariable analyses of TFS. Compared to patients with DPP-normal tumours, those with DPP-aberrant tumours had a HR of 2.12 (*p* < 0.0001; c-index 0.593; Fig. 3a) in univariable time-invariant analysis and a HR of 2.07 (*p* < 0.0001; Fig. 3b) in univariable time-dependent analysis. A statistically significant difference was observed in the c-indices when comparing the combined marker to the individual biomarkers of DNA ploidy (0.038; 95% CI 0.014–0.066; *p* = 0.0011) and PTEN (0.042; 95% CI 0.012–0.075; *p* = 0.0051).

In the subset of patients with GGG 1–2 tumours according to centrally reviewed Gleason scores, the HR for DPP-aberrant vs. DPP-normal was 1.99 (*p* < 0.0001; Supplementary Fig. S3A). The prognostic effect of the combined biomarker was not significantly different in the GGG 1–2 subgroup and the GGG 3–5 subgroup (*p* = 0.92; Supplementary Table S6; Supplementary Fig. S3).

The combined marker was more predictive of TFS when assessed using all blocks than using one random block per procedure (for univariable time-invariant analysis, HR 2.12 vs. mean HR 1.82; *p* < 0.0001 and c-index 0.593 vs. mean c-index 0.556; *p* < 0.0001; respectively; Supplementary Fig. S4).

In the subset of low-risk patients, the combined marker was associated with TFS with a HR for DPP-aberrant vs. DPP-normal tumours of 2.24 in time-invariant analysis (*p* = 0.0017; Fig. 3c) and 1.72 in time-dependent analysis (*p* = 0.019; Fig. 3d). In corresponding analyses including only intermediate-risk patients, the HR was 1.63 (*p* = 0.0086; Fig. 3e) and 1.89 (*p* = 0.0004; Fig. 3f), respectively. In time-invariant analyses, the estimated 5-year TFS for DPP-aberrant vs. DPP-normal tumours was 50% vs. 80% for low-risk patients and 32% vs. 52% for intermediate-risk patients. The corresponding 10-year TFS was 27% vs. 62% and 26% vs. 38%, respectively.

In time-invariant multivariable analysis including routine Gleason scores (Table 3), the combined biomarker was associated with TFS in analysis of all patients (HR 1.94; *p* < 0.0001) and patients diagnosed using biopsies (HR 2.02; *p* < 0.0001). Similar results were obtained in the corresponding analyses including centrally reviewed Gleason scores instead of routine Gleason scores (Supplementary Table S7).

**Table 3:** Uni- and multivariable analyses of treatment-free survival with patient characteristics at prostate cancer diagnosis, which include Gleason grade group based on routine Gleason scores.

Variable	Group		Univariable analysis		Multivariable analysis including patients diagnosed using biopsy^a^		Multivariable analysis including patients diagnosed using biopsy or TURP^b^	
		*N*	HR (95% CI)	*p* value	HR (95% CI)	*p* value	HR (95% CI)	*p* value
Combined DNA ploidy and PTEN	DPP-normal vs. DPP-aberrant	432	2.12 (1.58-2.85)	**<0.0001**	2.02 (1.46–2.81)	**<0.0001**	1.94 (1.42-2.67)	**<0.0001**
PSA		558		**0.0004**		**0.017**		**0.028**
	≤6 ng/ml	237	ref.		ref.		ref.	
	>6 ng/ml and ≤10 ng/ml	228	1.66 (1.24-2.22)		1.39 (0.96–2.02)		1.43 (1.00–2.04)	
	>10 ng/ml and ≤20 ng/ml	93	1.86 (1.27-2.72)		2.34 (1.31–4.20)		2.11 (1.20–3.71)	
Prostate volume	10-ml increment	506	0.85 (0.78-0.92)	**<0.0001**	0.82 (0.73–0.91)	**0.0004**	0.83 (0.75-0.93)	**0.0006**
PSA density	0.1-ng/ml/ml increment	506	1.11 (1.06-1.16)	**<0.0001**	0.98 (0.86–1.11)	0.7	0.99 (0.89-1.10)	0.86
Gleason grade group^c^	2 vs 1	558	2.41 (1.85-3.15)	**<0.0001**	2.28 (1.65–3.15)	**<0.0001**	2.30 (1.69-3.14)	**<0.0001**
Clinical T stage	cT2 vs cT0/1	558	1.42 (1.07-1.89)	**0.016**	1.22 (0.89–1.69)	0.22	1.27 (0.93–1.75)	0.14
Procedure type	Biopsy vs TURP	558	4.16 (2.42-7.15)	**<0.0001**	omitted		3.52 (1.87-6.64)	**0.0001**
Number of positive biopsy cores	1 increment	455	1.21 (1.12–1.31)	**<0.0001**	1.14 (1.04–1.26)	**0.0054**	omitted	
Age	10-year increment	558	1.10 (0.89-1.38)	0.38				

*CI* confidence interval, *DPP* normal DNA ploidy and PTEN “normal”, DPP-aberrant DNA ploidy and/or PTEN “aberrant”, *HR* hazard ratio, *PSA* prostate-specific antigen, *TURP* transurethral resection of the prostate. Values in bold are statistically significant at *p* = 0.05.

^a^Of the 558 patients, 339 (172 treated and 167 not treated) had complete data and were included in the multivariable analysis.

^b^Of the 558 patients, 400 (183 treated and 217 not treated) had complete data and were included in the multivariable analysis.

^c^Routine Gleason scoring performed according to the 2005 or 2014 International Society of Urological Pathology guidelines.

Patients with both non-diploid and PTEN loss tumours had higher HRs than those with only one of these aberrations, when compared to those with diploid and PTEN present tumours, in univariable time-invariant and time-dependent analyses (HR 2.98 vs. 2.13 and 3.10 vs. 1.88, respectively; Supplementary Fig. S5) and multivariable analyses of TFS (Supplementary Tables S8, S9). The c-index was 0.650 for the standard CAPRA score and 0.674 for the CAPRA score integrated with DNA ploidy and PTEN status in time-invariant analysis of TFS (Supplementary Table S10). The difference of 0.025 (95% CI 0.008–0.045) between these c-indices was significant (*p* = 0.0033). The difference in c-indices between the standard and the updated CAPRA score was 0.051 (95% CI 0.014–0.093; *p* = 0.0049) in low-risk and 0.032 (95% CI 0.002–0.064; *p* = 0.036) in intermediate-risk patients.

### DNA ploidy and PTEN status in analyses of treatment failure-free survival

Among 219 patients with available post-treatment follow-up data, DPP-aberrant tumours were detected in 72 (40%) of 180 patients with valid measurements at the diagnostic procedure, 110 (54%) of 203 patients with valid measurements. When considering all procedures with valid measurements collectively, DPP-aberrant tumours were found in at least one procedure for 135 (62%) of the 216 patients. The combined biomarker was associated with TFFS when evaluated across all procedures (HR 2.01; *p* = 0.027; Supplementary Fig. S6C), but not when assessed at the diagnostic procedure (*p* = 0.58; Supplementary Fig. S6A) or the last procedure before treatment (*p* = 0.29; Supplementary Fig. S6B).

## Discussion

The combined DNA ploidy and PTEN biomarker was consistently associated with TFS in univariable analyses, both those including only diagnostic procedures and those including all procedures performed before disease reclassification in a time-dependent manner. This association remained statistically significant in multivariable analyses including standard clinicopathologic characteristics. Furthermore, DNA ploidy and PTEN status improved risk stratification when integrated with the CAPRA score. This suggests that these biomarkers may contribute to identifying AS patients who are at risk of disease progression. Validating these results could potentially introduce novel biological criteria for AS, refining patient selection and optimizing the timing of curative treatment.

Incorporating DNA ploidy and PTEN status into the current AS protocol would result in curative treatment for an additional 81 (30%) of the 269 patients not treated before the end of follow-up and lead to earlier treatment for 97 (48%) of the 202 treated patients. We observed similar estimated 5-year TFS for low-risk patients with DPP-aberrant tumours and those with intermediate-risk with DPP-normal tumours. However, due to the more intensive monitoring in intermediate-risk cases, it is likely that low-risk patients with DPP-aberrant tumours would have worse TFS if monitored similarly. Additionally, the 10-year TFS of slightly above 25% suggests that low-risk patients with DPP-aberrant tumours might have TFS more similar to intermediate-risk patients with DPP-aberrant tumours than to intermediate-risk patients with DPP-normal tumours. Taken together, this indicates that most intermediate and low-risk patients with DPP-aberrant tumours will eventually be disease reclassified using the current AS protocol and could therefore potentially benefit from earlier initiation of treatment. This increase in curative treatment seems reasonable also given the twofold higher risk of treatment failure in patients with DPP-aberrant tumours. The main limitation of this study is the lack of information on longer-term outcomes, such as metastases and death, due to the low risk of progression and the extended follow-up required to capture such events in AS cohorts [7, 28]. Nevertheless, disease reclassification and treatment failure, as intermediate endpoints, are currently clinically relevant as they trigger subsequent therapeutic interventions.

The association between PCa outcomes and DNA ploidy or PTEN status as individual biomarkers has been well-documented in various studies conducted in both postoperative and preoperative settings [13–15]. Studies performed in watchful waiting cohorts suggest that patients with non-diploid or PTEN loss tumours are at higher risk of developing aggressive disease if left untreated, as these traits were associated with an increased risk of PCa-related mortality [23, 29, 30]. To date, only one study has investigated the prognostic value of PTEN in an AS cohort. Among the 190 low-risk patients included in that study, PTEN loss in the diagnostic biopsy correlated with TFS [10]. We have found no studies using DNA ploidy in the contemporary AS setting.

Previous studies involving RP specimens indicated that tumours characterized by both non-diploidy and PTEN loss might be more aggressive than those with only one of these aberrations [16, 31]. The findings of the present study also support this notion. However, due to the infrequent co-occurrence of non-diploidy and PTEN loss (5% of procedures), we implemented a dichotomous biomarker, grouping patients with either non-diploid tumours or PTEN loss, along with those with both aberrations. Importantly, the combined marker identified more patients at risk of disease reclassification than either biomarker individually, and provided higher prognostic value in terms of c-index.

The Gleason score is a robust prognostic indicator for oncologic outcomes in PCa patients, and the only tissue-based biomarker currently used in AS [6]. Disadvantages of Gleason score include considerable interobserver variability, and the fact that its prognostic value is highest when assessed by experienced uropathologists [32, 33]. This poses a particular challenge for patients receiving care at local hospitals, where sufficient level of expertise may not always be readily available. Following a central review of the slides using only the 2014 ISUP guidelines, 8% of the patients included in this study were reclassified to GGG 3–5 at diagnosis, which would typically render them ineligible for AS. Nonetheless, our combined marker showed similar HR in analysis of TFS regardless of whether including or excluding patients that were reclassified. Moreover, it retained statistical significance in multivariable analyses of TFS when including centrally reviewed GGG instead of routine GGG.

One of the strengths of the present study is the use of a community-based AS cohort with a well-defined protocol and comprehensive follow-up. As in other AS programmes, some patients were managed based on their personal preferences rather than adhering strictly to protocol-based criteria [6]. However, the prospective design of this study allowed us to identify these patients and they were therefore not included in our analyses to prevent introducing possible confounders. Our cohort’s inclusion criteria are one of the broadest among the published AS studies, incorporating 48% intermediate-risk patients, compared to 13–30% in previous AS reports [28, 34–36]. Nonetheless, the rate of disease reclassification (41% vs. 27-50%) [28, 35, 37, 38] and treatment failure fall within the range observed in previous studies (23% vs. 8–25%) [28, 36, 38, 39]. However, a notable limitation of our study is its reliance on a single AS cohort, underscoring the need for validation of our findings in cohorts from other hospitals.

Assessing tissue-based biomarkers in needle biopsies is challenged by limited tumour tissue, especially when blocks are already cut for routine histopathological examination. Overall, we successfully assessed DNA ploidy and PTEN status in 89% of the patients. However, our success rate could likely have been higher if the tissue sections for these measurements were cut in the routine histopathological workflow, as this would have resulted in less tissue loss during block trimming. The lowest fraction of valid measurements was for DNA ploidy analyses using monolayers, requiring the most tissue. Thus, we supplemented the DNA ploidy assessments from monolayers with those from tissue sections. An observed 93% agreement between DNA ploidy status measured using both methods suggests that DNA ploidy measurements can be reliably performed in the AS setting.

A consensus is lacking regarding the optimal dichotomization of PTEN scores in immunohistochemistry studies, with the most common threshold being 90% PTEN-positive cells [23, 24] In our previous study in two RP cohorts, including 87% and 67% intermediate or high-risk patients according to the postsurgical CAPRA (CAPRA-S) score, we used a 50% threshold for PTEN score categorization [16]. In this study, we opted for a 90% threshold, considering PTEN loss as an early event in PCa development [15] and potentially reduced sensitivity in detecting its reduced expression in needle biopsies. We averaged PTEN scores across all specimens for procedural assessment, as previously described in the RP study mentioned earlier [16], and in a study conducted on TURP [30], which analysed PTEN expression. However, an alternative based on the lowest score is also plausible.

It is well-established that extensive tumour heterogeneity in PCa negatively affects the assessment of tissue-based biomarkers [22, 40, 41]. To address this concern, we analysed DNA ploidy and PTEN in every available tumour-containing block from all procedures. Consistent with our previous findings [22], we observed heterogeneity in DNA ploidy in 22% diagnostic procedures. Notably, heterogeneity in all evaluated biomarkers increased by approximately one-third to nearly double across subsequent procedures, and it was more frequent in treated patients as compared to untreated individuals. This increase could indicate tumour growth, as we previously observed higher rates of heterogeneity in these biomarkers in more extensive tumours [22]. In the analyses of TFS, the combined biomarker had lower predictive value in terms of HRs and c-indices when using a single random specimen per procedure compared to compiling measurements from all specimens within each procedure. Furthermore, the combined marker only showed a statistically significant association with TFFS when assessed across all procedures. We, therefore, recommend considering tumour heterogeneity when assessing DNA ploidy and PTEN status in an AS setting. In practical terms, we suggest analysing all tissue sections from each biopsy procedure on the same slide whenever possible, to optimize time and reagent efficiency. For procedures with up to four positive needle biopsy cores, one slide could suffice, while cases with more than four positive needle biopsy cores may require the use of two to three slides.

Liquid-based biomarkers hold promise in addressing the challenges associated with tumour sampling in prostate needle biopsies, and have the potential to reduce the need for repeated biopsies. While no biomarkers have been specifically developed for men undergoing AS, several biomarkers primarily used in the screening and pre-diagnostic settings have been explored in this context. These biomarkers include the prostate health index blood test (PHI), which combines total PSA, free PSA, and [−2] proPSA, and the 4-kallikrein (4 K) blood test, which incorporates kallikrein-related peptidase 2 (hK2), intact PSA, free PSA, and total PSA, as well as the PCA3 assay, which detects prostate cancer antigen 3 (PCA3) transcript levels in urine. However, studies have indicated that while these tests can identify patients at risk of upgrading at the initial confirmatory biopsy, they did not provide any predictive value on subsequent surveillance biopsies [42–44]. This suggests that their ability to discriminate men with higher-risk disease may be limited to the initial patient selection. In contrast, our findings suggest that DNA ploidy and PTEN biomarkers, whether used alone or in combination, are associated with TFS both at diagnostic and surveillance biopsies.

In conclusion, our results indicate that DNA ploidy and PTEN status could aid in early identification of AS patients with high risk of progression. Our findings in AS align well with earlier results from studies of risk stratification following RP, thus indicating that DNA ploidy and PTEN may be used as biomarkers throughout the stages of PCa development and their different clinical handling. We recommend assessment of DNA ploidy and PTEN status in all available specimens to control for tumour heterogeneity and sparse sampling in AS.

### Supplementary information

Supplementary information

## Data Availability

The data generated in this study are not publicly available due to the potential privacy and consent concerns of research participants. De-identified individual patient-level data can be made available to other researchers upon reasonable request by contacting the corresponding author, subject to approval by the relevant people or review board at the institutions that provided the original data.

## Code availability

The code used to generate DNA ploidy classifications for tissue sections will be provided by the corresponding author upon a reasonable request.
