# Supplementary material for: DNA ploidy and PTEN as biomarkers for predicting aggressive disease in prostate cancer patients under active surveillance
Source: Br J Cancer. 2024 Jul 3;131(5):895–904. doi: 10.1038/s41416-024-02780-x (PMC11368925; doi:10.1038/s41416-024-02780-x)
Supplement: Supplementary file 1 — Supplementary information [file 41416_2024_2780_MOESM1_ESM.pdf]

Supplementary information

Table of Contents

DNA image cytometry on tissue sections 2
Figure S1: Patient status at the last follow-up (30th April 2023). 3
Figure S2: (a, c) Time-invariant and (b, d) time-dependent analyses of treatment-free survival grouped by (a, b) DNA ploidy or (c, d) PTEN status. 4
Figure S3: Time-invariant analyses of treatment-free survival grouped by the combined DNA ploidy and PTEN status. 5
Figure S4: Histograms of (a) hazard ratio (HR) in time-invariant analysis, (b) HR in time-dependent analysis, (c) c-index of the combined DNA ploidy and PTEN status in time-invariant analysis, and (d) c-index of CAPRA updated by adding 1 point if non-diploid and 1 if PTEN loss in time-invariant analysis, when calculated using one random block per procedure. 6
Figure S5: (a) Time-invariant and (b) time-dependent analyses of treatment-free survival grouped by the combined DNA ploidy and PTEN status as a three-group marker. 7
Figure S6: Time-invariant analyses of treatment failure-free survival, categorized based on the combined DNA ploidy and PTEN status assessed (A) at diagnosis, (B) at the last procedure before treatment, and (C) across all procedures. 8
Table S1: The Reporting Recommendations for Tumour Marker Prognostic Studies (REMARK) checklist 9
Table S2: Percentage of non-diploid and PTEN lost measurements at the patient, procedure, and block levels 11
Table S3: Patient characteristics in relation to the combined DNA ploidy and PTEN status at prostate cancer diagnosis 12
Table S4: Heterogeneity in DNA ploidy, PTEN status, and Gleason grade group among individual blocks within each procedure. 13
Table S5: Association between treatment and heterogeneity in DNA ploidy, PTEN status and Gleason grade group at the diagnostic procedure and the last procedure before any treatment. 14
Table S6: Multivariable time-invariant analysis of treatment-free survival with centralized review-based Gleason grade group, combined DNA ploidy and PTEN status, and their interaction term in the model.. 15
Table S7: Uni- and multivariable analyses of treatment-free survival with patient characteristics at prostate cancer diagnosis, which include Gleason grade group based on centrally reviewed Gleason scores 16
Table S8: Uni- and multivariable analyses of treatment-free survival with patient characteristics at prostate cancer diagnosis, which include combined DNA ploidy and PTEN status as a three-group biomarker and Gleason grade group based on routine Gleason scores 17
Table S9. Uni- and multivariable analyses of treatment-free survival with patient characteristics at prostate cancer diagnosis, which include combined DNA ploidy and PTEN status as a three-group biomarker and Gleason grade group based on centrally reviewed Gleason scores 18
Table S10: C-indices for PTEN status, DNA ploidy status and the combined DNA ploidy and PTEN status as well as the standard and updated CAPRA score at prostate cancer diagnosis in analyses of treatment-free survival. 19

DNA image cytometry on tissue sections

Tissue sections (5 µm) were stained using the Feulgen method. Tumour areas were marked by a pathologist (MP). Feulgen-stained nuclei in tissue sections were imaged by a Zeiss AxioImager microscope equipped with a green filter with a maximum at 546 nm and a 63x lens with a numerical aperture of 1.4. Nuclei were captured by FGrabber (Institute for Cancer Genetics and Informatics (ICGI), Oslo University Hospital (OUH), Norway), and separated from the background using the method described by Nielsen *et al*.[1] Identification of representative (e.g. intact, isolated and non-necrotic) epithelial nuclei and reference nuclei (i.e. lymphocytes and fibroblasts) was done automatically using the Nucleotyping Work Station (NWS) software (ICGI, OUH, Norway). All images of nuclei were verified by trained personnel who excluded images not representing a complete section of a single epithelial nucleus. DNA ploidy histograms were automatically classified into diploid and non-diploid (i.e. tetraploid or aneuploid) in the NWS software. Samples with fewer than 200 nuclei were considered indeterminate.

1 Nielsen B, Albregtsen F, Danielsen HE. Automatic segmentation of cell nuclei in Feulgen-stained histological sections of prostate cancer and quantitative evaluation of segmentation results. *Cytom A* 2012; **81 A**: 588–601.

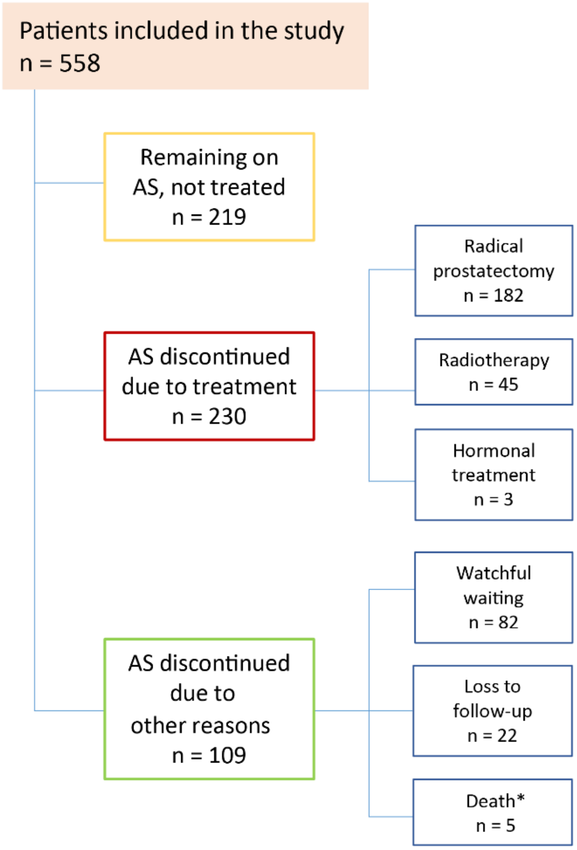

Figure S1: Patient status at the last follow-up (30th April 2023).
*None of the deaths were due to prostate cancer.

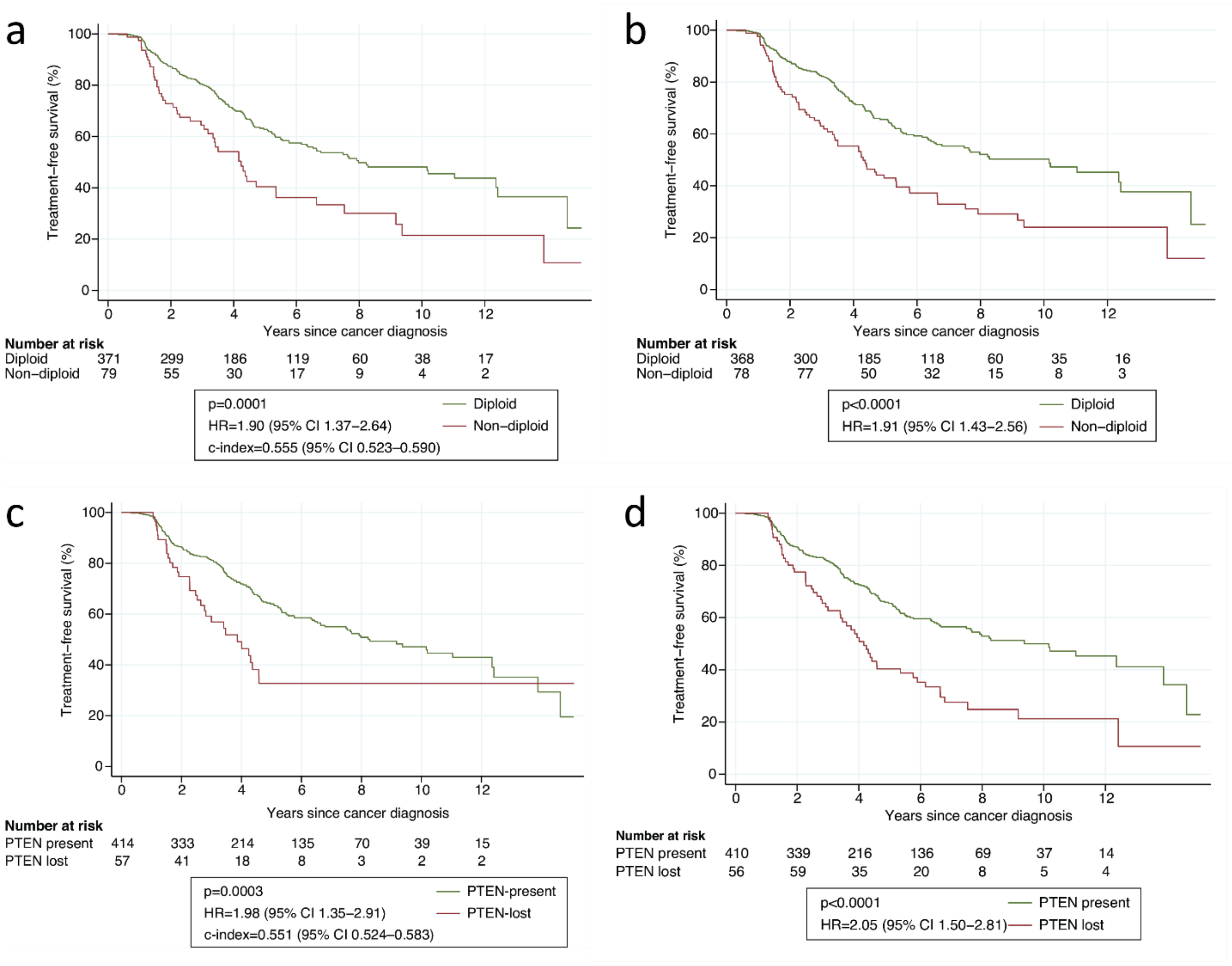

Figure S2: (a, c) Time-invariant and (b, d) time-dependent analyses of treatment-free survival grouped by (a, b) DNA ploidy or (c, d) PTEN status.
CI confidence interval, HR hazard ratio.

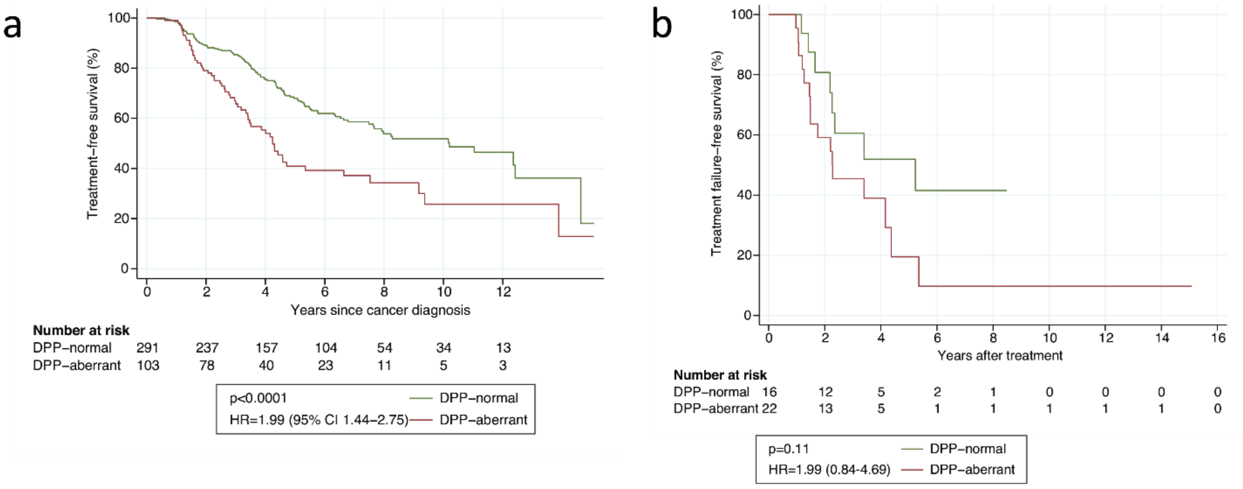

Figure S3: Time-invariant analyses of treatment-free survival grouped by the combined DNA ploidy and PTEN status. (a) Patients with Gleason grade group 1-2 tumours based on the centralized review. (b) Patients with Gleason grade group 3-5 tumours based on the centralized review.

CI confidence interval, HR hazard ratio.

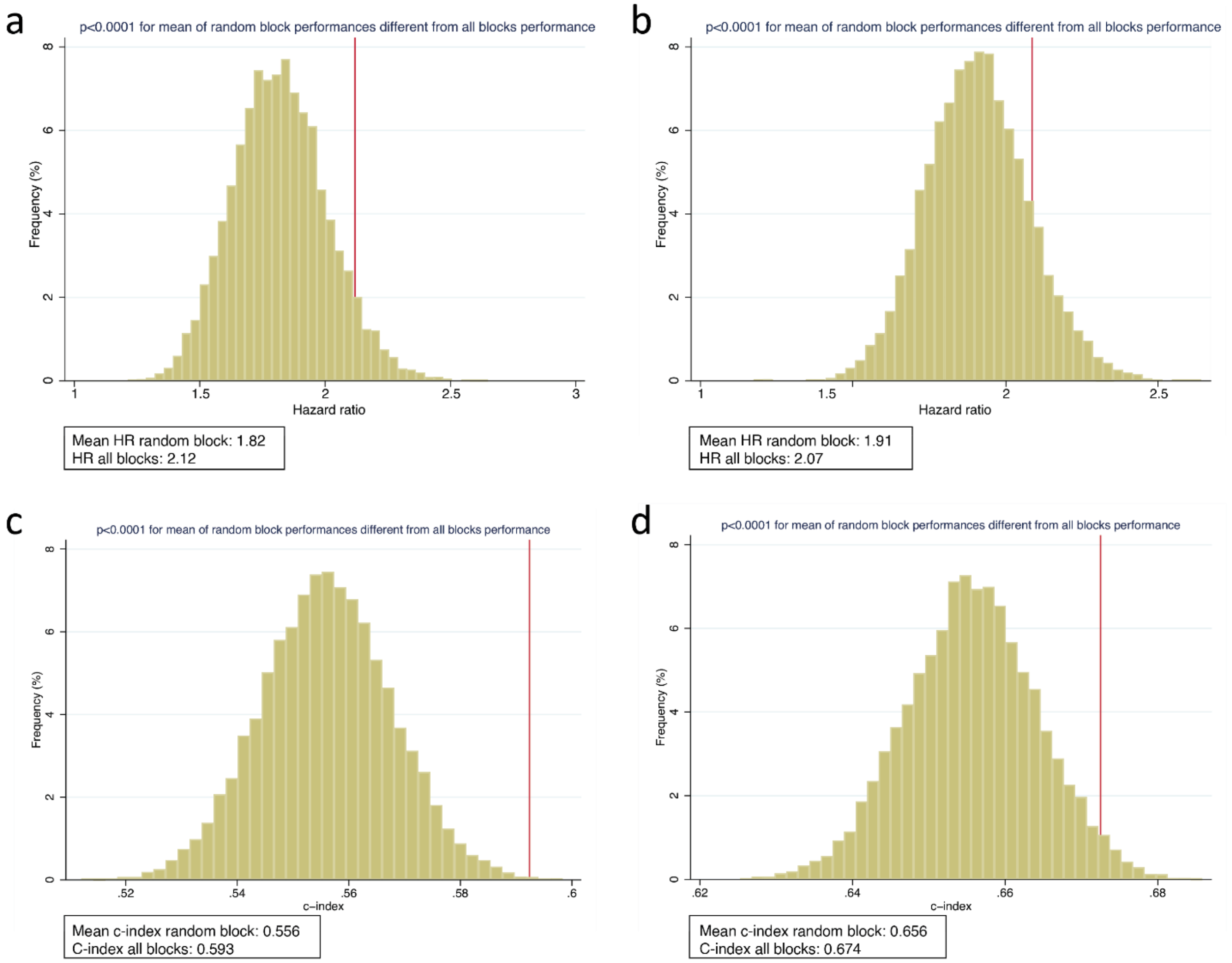

Figure S4: Histograms of (a) hazard ratio (HR) in time-invariant analysis, (b) HR in time-dependent analysis, (c) c-index of the combined DNA ploidy and PTEN status in time-invariant analysis, and (d) c-index of CAPRA updated by adding 1 point if non-diploid and 1 if PTEN loss in time-invariant analysis, when calculated using one random block per procedure. The red vertical line indicates the performance when using all blocks. The average of the observed performance for each of the 10,000 repeats with one random block per procedure was compared to the performance with all blocks using the one-sample t-test.

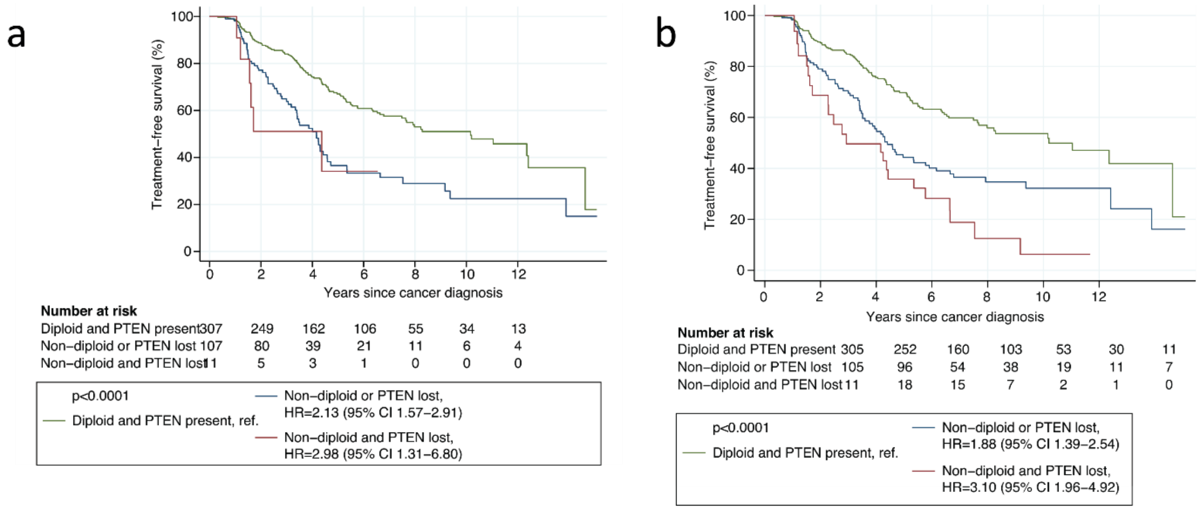

Figure S5: (a) Time-invariant and (b) time-dependent analyses of treatment-free survival grouped by the combined DNA ploidy and PTEN status as a three-group marker.
CI confidence interval, HR hazard ratio.

a

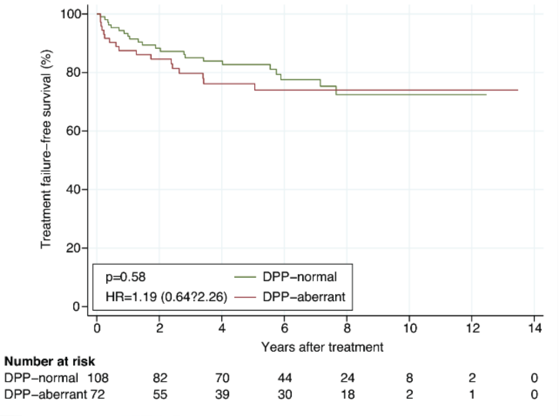

b

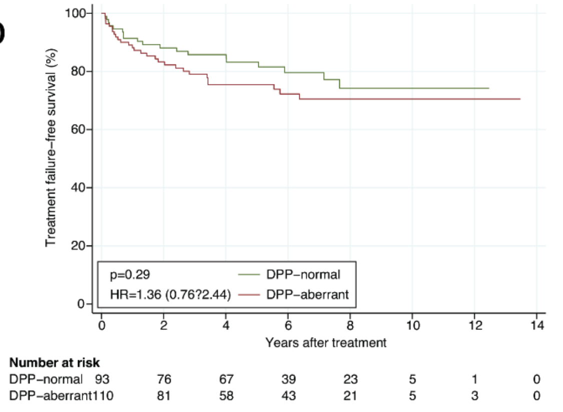

c

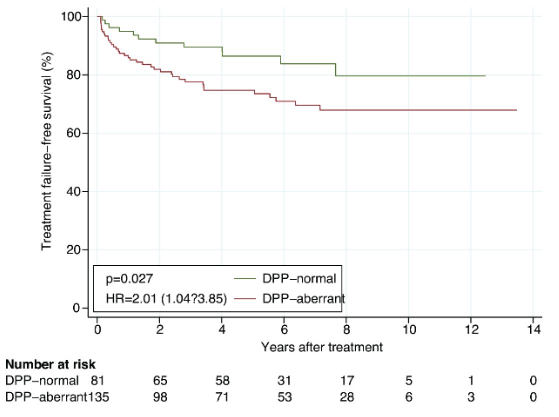

Figure S6: Time-invariant analyses of treatment failure-free survival, categorized based on the combined DNA ploidy and PTEN status assessed (A) at diagnosis, (B) at the last procedure before treatment, and (C) across all procedures.

CI confidence interval, DPP-normal DNA ploidy and PTEN “normal”, DPP-aberrant DNA ploidy and/or PTEN “aberrant”, HR hazard ratio.

Table S1: The Reporting Recommendations for Tumour Marker Prognostic Studies (REMARK) checklist

Item to be reported		Where reported	Comments
**INTRODUCTION**			
1	State the marker examined, the study objectives, and any pre-specified hypotheses.	Introduction, Patients and Methods	
**MATERIALS AND METHODS**			
Patients			
2	Describe the characteristics (e.g., disease stage or co-morbidities) of the study patients, including their source and inclusion and exclusion criteria.	Patients and Methods, Figure 1, Table 1	
3	Describe treatments received and how chosen (e.g., randomized or rule-based).	Patients and Methods, Results, Table 1	
Specimen characteristics			
4	Describe type of biological material used (including control samples) and methods of preservation and storage.	Patients and Methods	
Assay methods			
5	Specify the assay method used and provide (or reference) a detailed protocol, including specific reagents or kits used, quality control procedures, reproducibility assessments, quantitation methods, and scoring and reporting protocols. Specify whether and how assays were performed blinded to the study endpoint.	Patients and Methods, Supplementary information	
Study design			
6	State the method of case selection, including whether prospective or retrospective and whether stratification or matching (e.g., by stage of disease or age) was used. Specify the time period from which cases were taken, the end of the follow-up period, and the median follow-up time.	Patients and Methods, Results	
7	Precisely define all clinical endpoints examined.	Patients and Methods	
8	List all candidate variables initially examined or considered for inclusion in models.	Patients and Methods	
9	Give rationale for sample size; if the study was designed to detect a specified effect size, give the target power and effect size.		Included as many patients and samples as possible
Statistical analysis methods			
10	Specify all statistical methods, including details of any variable selection procedures and other model-building issues, how model assumptions were verified, and how missing data were handled.	Patients and Methods	
11	Clarify how marker values were handled in the analyses; if relevant, describe methods used for cutpoint determination.	Patients and Methods	
**RESULTS**			
Data			
12	Describe the flow of patients through the study, including the number of patients included in each stage of the analysis (a diagram may be helpful) and reasons for dropout. Specifically, both overall and for each subgroup extensively examined report the numbers of patients and the number of events.	Patients and Methods, Figure 1, Figure S1, Table 1, Results, Figure 3, Figure 4, Table 3, Figure S2, Table S5-S7	
13	Report distributions of basic demographic characteristics (at least age and sex), standard (disease-specific)	Results, Table 1	

	prognostic variables, and tumor marker, including numbers of missing values.		
Analysis and presentation			
14	Show the relation of the marker to standard prognostic variables.	Results, Table S3	
15	Present univariable analyses showing the relation between the marker and outcome, with the estimated effect (e.g., hazard ratio and survival probability). Preferably provide similar analyses for all other variables being analyzed. For the effect of a tumor marker on a time-to-event outcome, a Kaplan-Meier plot is recommended.	Table 3, Table S5-S7, Figure 3, Figure 4, Figure S2, Figure S3, Figure S5,	
16	For key multivariable analyses, report estimated effects (e.g., hazard ratio) with confidence intervals for the marker and, at least for the final model, all other variables in the model.	Table 3, Table S5-S7	
17	Among reported results, provide estimated effects with confidence intervals from an analysis in which the marker and standard prognostic variables are included, regardless of their statistical significance.	Table 3, Table S5-S7	Included variables that were significant in univariable analysis of treatment-free survival.
18	If done, report results of further investigations, such as checking assumptions, sensitivity analyses, and internal validation.	Patients and Methods, Figure S3, Table 3, Table S5-S7	We conducted tests to assess the proportional-hazards assumption and performed subgroup analyses for treatment-free survival.
**DISCUSSION**			
19	Interpret the results in the context of the pre-specified hypotheses and other relevant studies; include a discussion of limitations of the study.	Discussion	
20	Discuss implications for future research and clinical value.	Discussion	

Table S2: Percentage of non-diploid and PTEN lost measurements at the patient, procedure, and block levels

Measurement	Patient level	Procedure level	Block level
**Non-diploid**	33 (167/501)	23 (221/978)	15 (348/2393)
**PTEN lost**	23 (118/520)	16 (160/1030)	13 (334/2663)
**Non-diploid and PTEN lost**	10 (47/493)	5 (51/943)	3 (60/2189)
**Non-diploid and/or PTEN lost**	45 (221/495)	34 (330/958)	27 (622/2264

The data is shown as percentage (count/total).

Table S3: Patient characteristics in relation to the combined DNA ploidy and PTEN status at prostate cancer diagnosis

Characteristic	DPP-normal	DPP-aberrant	p-value[d]
**Patients**	307	125	
**Age – yr**	65 (61-69)	66 (61-71)	0.082
**PSA**			0.43
≤6 ng/ml	141 (46)	49 (39)	
>6 ng/ml and ≤10 ng/ml	118 (38)	55 (44)	
>10 ng/ml and ≤20 ng/ml	48 (16)	21 (17)	
**Prostate volume – ml**	40 (30-56)	43 (30-55)	0.84
Missing	24 (8)	8 (6)	
**PSA density – ng/ml/cm^3**	0.16 (0.11-0.22)	0.17 (0.12-0.24)	0.18
Missing	24 (8)	8 (6)	
**Gleason grade group[a]**			**<0.0001**
1	217 (71)	59 (47)	
2	90 (29)	66 (53)	
**Gleason grade group[b]**			**<0.0001**
1	145 (47)	26 (21)	
2	146 (48)	77 (62)	
3	9 (3)	11 (9)	
4	3 (1)	8 (6)	
5	2 (1)	1 (1)	
Missing	2 (1)	2 (2)	
**Clinical T stage**			**0.036**
cT0/pT1	226 (74)	79 (63)	
cT2	81 (26)	46 (37)	
**CAPRA score[c]**			**0.0006**
0–2	187 (61)	53 (42)	
3–5	120 (39)	72 (58)	
Missing	0	0	
**Percentage of positive biopsy cores**	17 (10-25)	30 (17-50)	**<0.0001**
Missing	5 (2)	2 (1)	
**Maximum tumour extent in biopsy cores – mm**	4.0 (2.0-6.0)	6.0 (3.9-8.0)	**<0.0001**
Missing	2 (1)	3 (2)	

The data is shown as median (interquartile range) or number (percentage). Due to rounding the numbers may not sum to 100%. CAPRA Cancer of the Prostate Risk Assessment, DPP-normal DNA ploidy and PTEN "normal", DPP-aberrant DNA ploidy and/or PTEN "aberrant", PSA prostate-specific antigen, TURP transurethral resection of the prostate.

[a]Routine Gleason scoring performed according to the 2005 or 2014 International Society of Urological Pathology guidelines.

[b]Centrally reviewed Gleason scoring performed according to the 2014 International Society of Urological Pathology guidelines.

[c]When computing CAPRA score for patients diagnosed with TURP, the percentage of positive biopsies was replaced by the tumour percentage in the TURP specimen; specifically, 0 points were assigned for <5% tumour and 1 point was assigned for ≥5% tumour. Computation of CAPRA score was performed using routinely assessed Gleason scores.

[d]Fisher's exact (categorical variables) or Mann–Whitney's *U* (continuous variables) test were used to evaluate associations.

Table S4: Heterogeneity in DNA ploidy, PTEN status, and Gleason grade group among individual blocks within each procedure.

Variable	Diagnostic procedure	1st surveillance procedure	2nd surveillance procedure	3rd surveillance procedure
**DNA ploidy status (diploid vs non-diploid)**	22 (52/240)	26 (54/207)	32 (35/110)	38 (14/37)
**PTEN status (PTEN present vs PTEN lost)**	14 (40/285)	22 (52/233)	21 (25/117)	25 (9/36)
**Combined DNA ploidy and PTEN status (DPP-normal vs DPP aberrant)**	31 (71/229)	37 (74/198)	41 (44/107)	45 (15/33)
**Gleason grade group (GGG 1-5)[a]**	51 (169/335)	62 (161/260)	58 (80/137)	70 (29/41)

Only patients with a minimum of two tumour-containing tissue blocks yielding valid results for a specific marker during a particular procedure were considered for the calculations.
The data is shown as percentage (count/total).
DPP-normal DNA ploidy and PTEN "normal", DPP-aberrant DNA ploidy and/or PTEN "aberrant",
[a]Centrally reviewed Gleason scoring performed according to the 2014 International Society of Urological Pathology guidelines.

Table S5: Association between treatment and heterogeneity in DNA ploidy, PTEN status and Gleason grade group at the diagnostic procedure and the last procedure before any treatment.

Characteristic	Not treated	Treated	p-value*
**Patients**	328	230	
**Diagnostic procedure**			
**DNA ploidy**			0.35
Homogeneous	98 (30)	90 (39)	
Heterogeneous	23 (7)	29 (12)	
Missing	207 (63)	111 (48)	
**PTEN status**			
Homogeneous	136 (41)	109 (47)	**0.041**
Heterogeneous	15 (5)	25 (11)	
Missing	177 (54)	96 (42)	
**Combined DNA ploidy and PTEN status**			
Homogeneous	87 (27)	71 (31)	**0.033**
Heterogeneous	28 (9)	43 (19)	
Missing	213 (65)	166 (50)	
**Gleason grade group**[a]			**0.048**
Homogeneous	101 (31)	65 (28)	
Heterogeneous	84 (26)	85 (37)	
Missing	143 (44)	80 (35)	
**Last procedure before any treatment**			
**DNA ploidy**			**0.0017**
Homogeneous	85 (26)	112 (49)	
Heterogeneous	22 (7)	70 (30)	
Missing	221 (67)	48 (21)	
**PTEN status**			**0.0057**
Homogeneous	115 (35)	132 (57)	
Heterogeneous	19 (6)	50 (22)	
Missing	194 (59)	48 (21)	
**Combined DNA ploidy and PTEN status**			**0.0009**
Homogeneous	77 (24)	95 (41)	
Heterogeneous	27 (8)	81 (35)	
Missing	224 (68)	54 (24)	
**Gleason grade group**[a]			**<0.0001**
Homogeneous	72 (22)	153 (67)	
Heterogeneous	77 (23)	52 (23)	
Missing	178 (54)	25 (11)	

The data is shown as number (percentage). Due to rounding the numbers may not sum to 100%.

[a]Centrally reviewed Gleason scoring performed according to the 2014 International Society of Urological Pathology guidelines.

Table S6: Multivariable time-invariant analysis of treatment-free survival with centralized review-based Gleason grade group, combined DNA ploidy and PTEN status, and their interaction term in the model

Variable	Group	HR (95% CI)	p-value
**Gleason grade group**	1-2 vs 3-5	1.97 (0.96-4.06)	0.065
**Combined DNA ploidy and PTEN**	DPP-normal vs DPP-aberrant	1.98 (1.43-2.74)	<0.0001
**Gleason grade group/ Combined DNA ploidy and PTEN interaction**		0.95 (0.38-2.38)	0.92

CI confidence interval, DPP-normal DNA ploidy and PTEN “normal”, DPP-aberrant DNA ploidy and/or PTEN “aberrant”, HR hazard ratio.

Table S7: Uni- and multivariable analyses of treatment-free survival with patient characteristics at prostate cancer diagnosis, which include Gleason grade group based on centrally reviewed Gleason scores

Variable	Group	Univariable analysis			Multivariable analysis including patients diagnosed using biopsy[a]		Multivariable analysis including patients diagnosed using biopsy or TURP[b]	
		N	HR (95% CI)	p-value	HR (95% CI)	p-value	HR (95% CI)	p-value
**Combined DNA ploidy and PTEN**	DPP-normal vs DPP-aberrant	432	2.12 (1.58-2.85)	**<0.0001**	2.23 (1.60-3.10)	**<0.0001**	2.09 (1.51-2.89)	**<0.0001**
**PSA**		558		**0.0004**		**0.0022**		**0.0036**
	≤6 ng/ml	237	ref.		ref.		ref.	
	>6 ng/ml and ≤10 ng/ml	228	1.66 (1.24-2.22)		1.57 (1.08-2.28)		1.61 (1.13-2.31)	
	>10 ng/ml and ≤20 ng/ml	93	1.86 (1.27-2.72)		2.86 (1.58-5.18)		2.54 (1.44-4.51)	
**Prostate volume**	10-ml increment	506	0.85 (0.78-0.92)	**<0.0001**	0.82 (0.73-0.92)	**0.0007**	0.84 (0.76-0.93)	**0.0014**
**PSA density**	0.1-ng/ml/ml increment	506	1.11 (1.06-1.16)	**<0.0001**	0.96 (0.85-1.10)	0.58	0.98 (0.88-1.10)	0.78
**Gleason grade group[c]**		528		**<0.0001**		**0.0017**		**0.0002**
	1	230	ref.		ref.		ref.	
	2	257	1.96 (1.46-2.62)		1.40 (0.97-2.04)		1.59 (1.12-2.26)	
	3	25	4.24 (2.52-7.14)		2.26 (1.20-4.26)		2.73 (1.49-5.01)	
	4	13	5.46 (2.61-11.45)		3.30 (1.43-7.60)		3.78 (1.65-8.67)	
	5	3	5.97 (1.45-24.56)		7.91 (1.86-33.62)		7.12 (1.68-30.09)	
**Clinical T stage**	cT2 vs cT0/1	558	1.42 (1.07-1.89)	**0.016**	1.16 (0.84-1.60)	0.37	1.20 (0.87-1.65)	0.27
**Procedure type**	Biopsy vs TURP	558	4.16 (2.42-7.15)	**<0.0001**	omitted		2.71 (1.43-5.13)	**0.0022**
**Number of positive biopsy cores**	1 increment	455	1.21 (1.12-1.31)	**<0.0001**	1.14 (1.04-1.25)	**0.0068**	omitted	
**Age**	10-year increment	558	1.10 (0.89-1.38)	0.38				

CI confidence interval, DPP-normal DNA ploidy and PTEN “normal”, DPP-aberrant DNA ploidy and/or PTEN “aberrant”, HR hazard ratio; PSA prostate-specific antigen, TURP transurethral resection of the prostate,

[a]Of the 558 patients, 337 (171 treated and 166 not treated) had complete data and were included in the multivariable analysis.

[b]Of the 558 patients, 397 (182 treated and 215 not treated) had complete data and were included in the multivariable analysis.

[c]Centrally reviewed Gleason scoring performed according to the 2014 International Society of Urological Pathology guidelines.

Table S8: Uni- and multivariable analyses of treatment-free survival with patient characteristics at prostate cancer diagnosis, which include combined DNA ploidy and PTEN status as a three-group biomarker and Gleason grade group based on routine Gleason scores

Variable	Group	Univariable analysis			Multivariable analysis including patients diagnosed using biopsy[a]		Multivariable analysis including patients diagnosed using biopsy or TURP[b]	
		N	**HR (95% CI)**	**p-value**	**HR (95% CI)**	**p-value**	**HR (95% CI)**	**p-value**
**Combined DNA ploidy and PTEN**		425		**<0.0001**		**<0.0001**		**<0.0001**
	Diploid and PTEN>90%	307	ref.		ref.		ref.	
	Non-diploid or PTEN≤90%	107	2.13 (1.57-2.91)		2.05 (1.46-2.88)		1.96 (1.41-2.73)	
	Non-diploid and PTEN≤90%	11	2.98 (1.31-6.80)		3.09 (1.32-7.25)		3.43 (1.48-7.96)	
**PSA**		558		**0.0004**		**0.034**		0.055
	≤6 ng/ml	237	ref.		ref.		ref.	
	>6 ng/ml and ≤10 ng/ml	228	1.66 (1.24-2.22)		1.38 (0.95-2.01)		1.41 (0.98-2.02)	
	>10 ng/ml and ≤20 ng/ml	93	1.86 (1.27-2.72)		2.20 (1.21-3.99)		1.97 (1.11-3.49)	
**Prostate volume**	10-ml increment	506	0.85 (0.78-0.92)	**<0.0001**	0.82 (0.73-0.91)	**0.0003**	0.84 (0.76-0.93)	**0.0006**
**PSA density**	0.1-ng/ml/ml increment	506	1.11 (1.06-1.16)	**<0.0001**	0.98 (0.86-1.11)	0.73	1.00 (0.89-1.11)	0.93
**Gleason grade group[c]**	2	558	2.41 (1.85-3.15)	**<0.0001**	2.41 (1.74-3.35)	**<0.0001**	2.43 (1.78-3.31)	**<0.0001**
**Clinical T stage**	cT2 vs cT0/1	558	1.42 (1.07-1.89)	**0.016**	1.20 (0.87-1.66)	0.27	1.25 (0.91-1.72)	0.18
**Procedure type**	Biopsy vs TURP	558	4.16 (2.42-7.15)	**<0.0001**	omitted		3.60 (1.91-6.80)	**0.0001**
**Number of positive biopsy cores**	1 increment	455	1.21 (1.12-1.31)	**<0.0001**	1.13 (1.03-1.24)	**0.014**	omitted	
**Age**	10-year increment	558	1.10 (0.89-1.38)	0.38				

CI confidence interval, HR hazard ratio, PSA prostate-specific antigen, TURP transurethral resection of the prostate.

[a]Of the 558 patients, 332 (169 treated and 163 not treated) had complete data and were included in the multivariable analysis.

[b]Of the 558 patients, 393 (180 treated and 213 not treated) had complete data and were included in the multivariable analysis.

[c]Routine Gleason scoring performed accoring to the 2005 or 2014 International Society of Urological Pathology guidelines.

Table S9. Uni- and multivariable analyses of treatment-free survival with patient characteristics at prostate cancer diagnosis, which include combined DNA ploidy and PTEN status as a three-group biomarker and Gleason grade group based on centrally reviewed Gleason scores

Variable	Group		Univariable analysis		Multivariable analysis including patients diagnosed using biopsy[a]		Multivariable analysis including patients diagnosed using biopsy or TURP[b]	
		N	HR (95% CI)	p-value	HR (95% CI)	p-value	HR (95% CI)	p-value
**Combined DNA ploidy and PTEN**		425		**<0.0001**		**<0.0001**		**<0.0001**
	Diploid and PTEN>90%	307	ref.		ref.		ref.	
	Non-diploid or PTEN≤90%	107	2.13 (1.57-2.91)		2.31 (1.64-3.25)		2.15 (1.54-3.00)	
	Non-diploid and PTEN≤90%	11	2.98 (1.31-6.80)		2.20 (0.91-5.33)		2.54 (1.07-6.01)	
**PSA**		558		**0.0004**		**0.0061**		**0.0087**
	≤6 ng/ml	237	ref.		ref.		ref.	
	>6 ng/ml and ≤10 ng/ml	228	1.66 (1.24-2.22)		1.53 (1.05-2.23)		1.58 (1.10-2.27)	
	>10 ng/ml and ≤20 ng/ml	93	1.86 (1.27-2.72)		2.66 (1.45-4.88)		2.38 (1.33-4.27)	
**Prostate volume**	10-ml increment	506	0.85 (0.78-0.92)	**<0.0001**	0.82 (0.73-0.93)	**0.0012**	0.85 (0.76-0.94)	**0.0025**
**PSA density**	0.1-ng/ml/ml increment	506	1.11 (1.06-1.16)	**<0.0001**	0.97 (0.85-1.10)	0.62	0.99 (0.88-1.11)	0.84
**Gleason grade group**[c]		528		**<0.0001**		**0.0028**		**0.0005**
	1	230	ref.		ref.		ref.	
	2	257	1.96 (1.46-2.62)		1.40 (0.96-2.04)		1.59 (1.12-2.26)	
	3	25	4.24 (2.52-7.14)		2.18 (1.12-4.23)		2.55 (1.35-4.82)	
	4	13	5.46 (2.61-11.45)		3.21 (1.40-7.40)		3.66 (1.60-8.41)	
	5	3	5.97 (1.45-24.56)		7.79 (1.83-33.11)		7.05 (1.67-29.80)	
**Clinical T stage**	cT2 vs cT0/1	558	1.42 (1.07-1.89)	**0.016**	1.15 (0.83-1.59)	0.42	1.18 (0.85-1.63)	0.32
**Procedure type**	Biopsy vs TURP	558	4.16 (2.42-7.15)	**<0.0001**	omitted		2.78 (1.47-5.26)	**0.0017**
**Number of positive biopsy cores**	1 increment	455	1.21 (1.12-1.31)	**<0.0001**	1.14 (1.03-1.25)	**0.0088**	omitted	
**Age**	10-year increment	558	1.10 (0.89-1.38)	0.38				

CI confidence interval, HR hazard ratio, PSA prostate-specific antigen, TURP transurethral resection of the prostate.
[a]Of the 558 patients, 330 (168 treated and 162 not treated) had complete data and were included in the multivariable analysis.
[b]Of the 558 patients, 390 (179 treated and 211 not treated) had complete data and were included in the multivariable analysis.
[c]Centrally reviewed Gleason scoring performed according to the 2014 International Society of Urological Pathology guidelines.

Table S10: C-indices for PTEN status, DNA ploidy status and the combined DNA ploidy and PTEN status as well as the standard and updated CAPRA score at prostate cancer diagnosis in analyses of treatment-free survival.

Characteristic	c-index (95% CI)		
	All patients	Low risk	Intermediate risk
**DNA ploidy status**	0.555 (0.523-0.590)	0.557 (0.523-0.590)	0.539 (0.497-0.585)
**PTEN status**	0.551 (0.524-0.583)	0.557 (0.514-0.616)	0.537 (0.504-0.577)
**Combined DNA ploidy and PTEN**	0.593 (0.556-0.630)	0.601 (0.542-0.665)	0.562 (0.513-0.610)
**CAPRA score**	0.650 (0.611 to 0.686)	0.573 (0.504 to 0.631)	0.549 (0.496 to 0.602)
**CAPRA score integrated with the combined DNA ploidy and PTEN**	0.674 (0.636 to 0.710)	0.622 (0.544 to 0.690)	0.580 (0.524 to 0.637)

Calculations were performed only for patients with complete data on PTEN, DNA ploidy, combined DNA ploidy and PTEN status, and CAPRA score.

CAPRA Cancer of the Prostate Risk Assessment, CI confidence interval.
